# Book Reviews

**Published:** 1905-10

**Authors:** 


### BOOK REVIEWS.

THE DIAGNOSTICS OF INTERNAL MEDICINE. By Glenworth Reeve Butler, Sc. D., M. D., Chief of the Second Medical Division, Methodist Episcopal Hospital; Attending Physician to the Brooklyn Hospital; Consulting Physician to the Bushwick Central Hospital, etc.

The most important question in the treatment of disease is a correct diagnosis. This is the fundamental principle underlying scientific medicine, for without it the very best treatment may be misdirected. The "Diagnostics of Internal Medicine" by Butler is convenient, comprehensive and thorough. It gives the physician a hint as to the possible diseases of every region of the body by a series of artistic photographs with markings in the various places, which show the points where pain is likely to be felt. By exclusion and reference to the symptoms and diagnoses in the latter part of the book the significance of the pains may be determined. Full and valuable instructions are given as to taking histories and physical examinations.

All the approved methods of obtaining a correct diagnosis by microscopic examinations of the urine, blood, sputum, feces, etc., are given in detail, making this a most valuable book on diagnosis.

---

MANUAL OF DISEASES OF THE EYE.—By Charles H. May. 400 pages. 21 colored plates, including 60 colored figures. 360 engravings in the text. Fourth edition. Price, muslin $2.00 net. William Wood & Company, 51 Fifth Ave., New York.

The practical value of this book won for it an enviable place among the text-books for the student and general practitioner from its first edition in 1900.

Since then three editions have been required to supply the demand. This is sufficient evidence of the favor with which it has been received. The value of the work of Dr. May lies in the prominence given to important matters by omitting "excessive detail."

This enables the casual reader to grasp the essential features, many of which might be overlooked in more extensive discussions. Specialists in this line would probably prefer a more comprehensive work; this however does not detract from the book, as its chief aim is to supply a book for the student and general practitioner.

---

JACKSON ON THE SKIN. A Ready Reference Hand-book on Diseases of the Skin. By George Thomas Jackson, M. D., Chief of Clinic and Instructor in Dermatology, College of Physicians and Surgeons (Columbia University), New York. Fifth edition, enlarged and thoroughly revised. In one 12mo volume of 676 pages with 91 engravings and 3 colored plates. Cloth, $2.75 net. Lea Brothers & Co., Publishers, Philadelphia and New York, 1905.

This edition shows a continuance of the terse and practical style of instruction and alphabetical arrangement of subjects making its predecessors. The subjects are treated sufficiently clearly and the volume is of sufficient brevity to render it continuously a most useful reference-book.

---

### THE COUNCIL ON PHARMACY AND CHEMISTRY REPORTS ON ACETANILID MIXTURES.

The first official publication made by the Council on Pharmacy and chemistry appears in the *Journal A. M. A.*, May 27. A subcommittee has purchased in the open market original sealed packages of various proprietary and patent remedies containing acetanilid—some of them represented to be chemical compounds and publishes results of its examination of them as follows: Ammonol: acetanilid, 50; sodium bicarb., 25; ammonium carb., 20, antikamnia: acetanilid, 68; caffein, 5; citric acid, 5; sodium bicarb., 20. Koehler's headache powders: acetanilid, 76; caffein, 22. Orangeine: acetanilid, 43; sodium bicarb., 20; caffein, 10. Phenalgin: acetanilid, 57; sodium bicarb., 29; ammonium carb., 10. Salacetin: acetanilid, 43; sodium bicarb., 21; sodium salicylate, 20. All of these were mixtures. The *Journal*, commenting on the work, calls attention to the danger involved in the physician's prescribing such a remedy as one of the above without knowing that it contains acetanilid. The report is extremely conservative, and repeated analyses have been made so that no erroneous statement should be published.

